# Case Report: Splenic Ischemia Induced by Volvulus of Ileum

**DOI:** 10.3389/fsurg.2022.888332

**Published:** 2022-06-01

**Authors:** Mauro Sergi, Giulia Chisari, Sofia Paolo Lombardo, Paolo Fontana

**Affiliations:** ^1^ Department of Surgery, University Hospital, Catania, Italy; ^2^ Department of Experimental Oncology, Mediterranean institute of Oncology, Viagrande, Italy

**Keywords:** CT scan, volvulus, splenic ischemia, whirlpool sign, acute abdomen

## Abstract

A 31-year-old female patient was admitted to the emergency department with signs and symptoms of acute abdomen. Urgent CT scan was performed and small bowel volvulus, with whirlpool sign, was noted and torsion of the spleen was also involved too.

## Introduction

Volvulus is a special form of mechanical intestinal obstruction. It results from abnormal twisting of a loop of bowel around the axis of its own mesentery (1).

The involved segment of the bowel may be either completely or partially occluded with associated arterial or venous occlusion. The most common site for volvulus is the colon (3). Small bowel volvulus is rare and only a few cases have been reported worldwide. Volvulus of the small bowel accounts for <7% of all cases of small bowel obstruction (2, 3).

Our case underlines the importance of the differential diagnosis in the splenic artery ischemia where the implication of small bowel volvulus must be considered.

## Case Report

**Figure 1:**
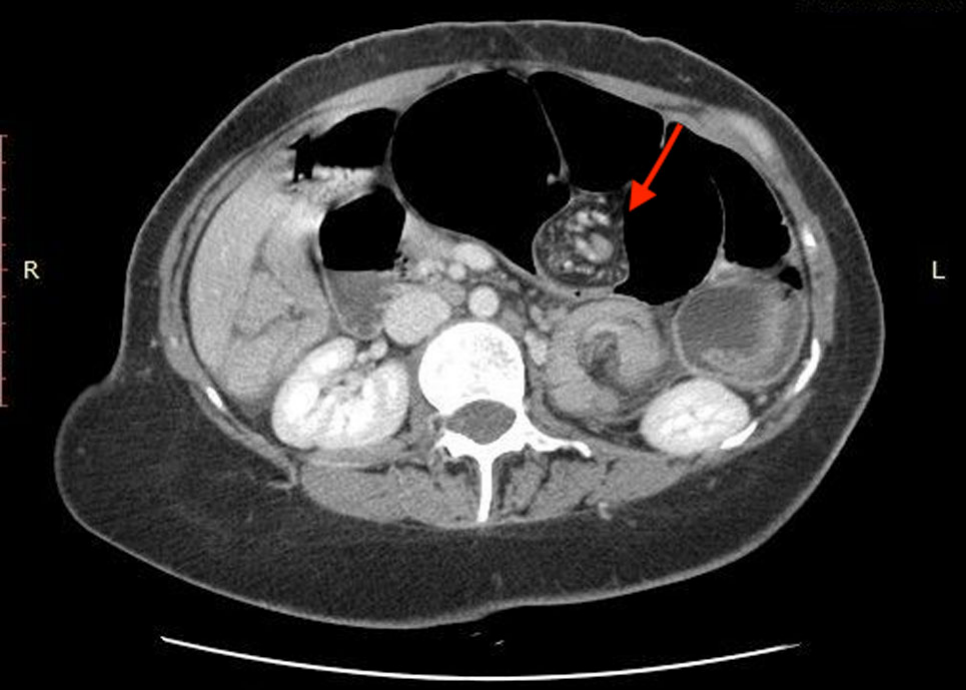
“Whirl” radiological sign of volvulus, involving mesenteric fat, pancreas’s tail, and the splenic venous axis.

**Figure 2:**
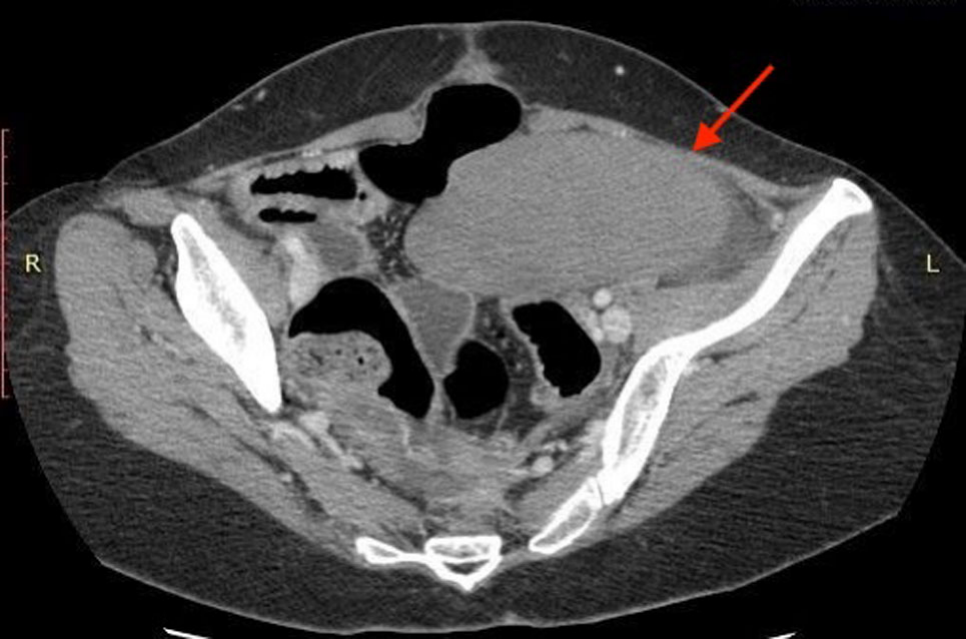
Increased spleen size, caudalized and with reduced enhancement after MDC. Noted also intra-abdominal overflow.

**Figure 3:**
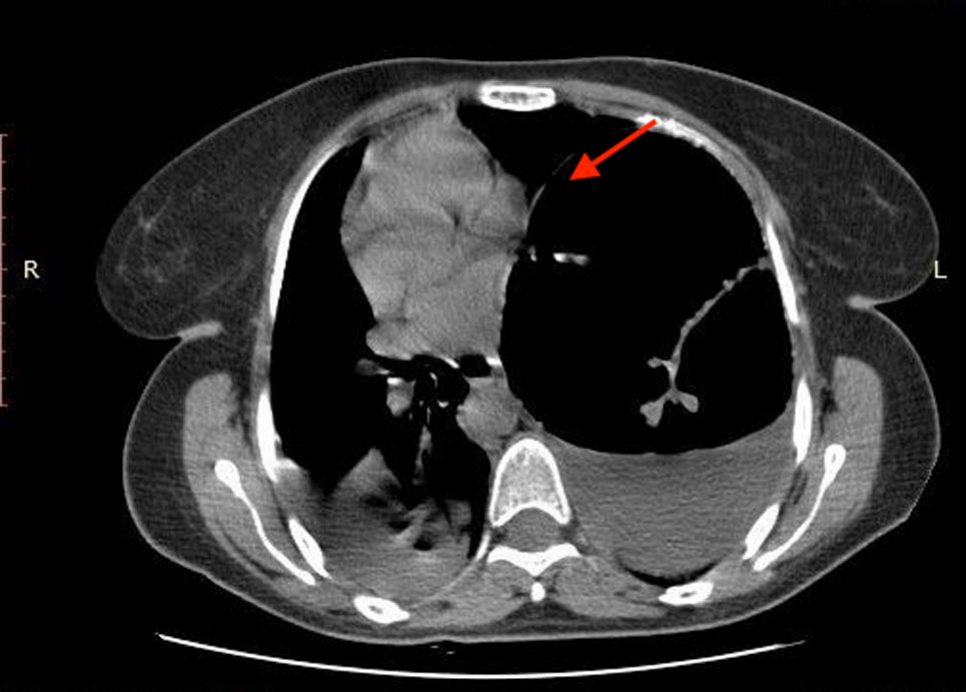
Marked overdistension of loops with contralateral dislocation of heart and middle-inferior mediastinum.

**Figure 4:**
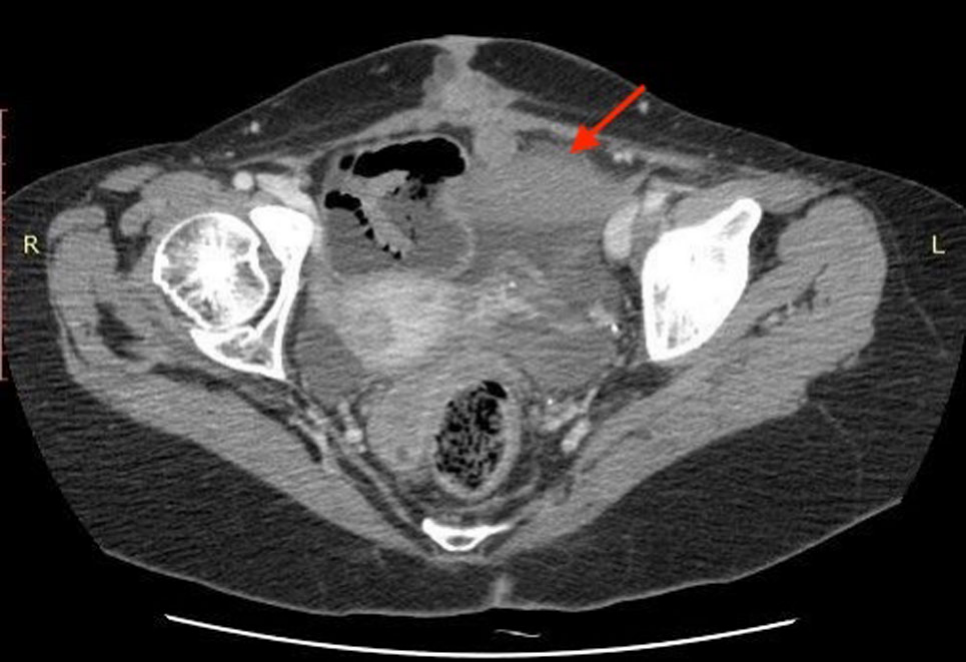
Median sub-umbilical laparocele.

We report the case of a 31-year-old female patient with a 3-day history of abdominal distension and associated abdominal pain. Her past medical history was relevant for total colectomy with ileo-rectal anastomosis due to recurrent volvuli. Laparocele of the lower abdomen region was observed. Blood pressure was stable with the presence of tachycardia. Blood investigations revealed no abnormal findings. Abdominal radiographs demonstrated dilated small bowels with fluid levels compatible with intestinal obstruction. Plain CT scan showed marked over-distension of the small bowel with air-fluid levels in the mesentery extending up to the left abdominal quadrant. The ileum appeared twisted around his vascular pedicle matching the “whirl” radiological sign of volvulus (Figure 1). The mesenteric fat, the tail portion of the pancreas and abdominal visceral vessels, with reference to the vascular splenic arteries were involved. After intravenous contrast administration, the splenic artery still showed enhancement, while no enhancement of the splenic vascular system was observed. Spleen size was increased but showed reduced enhancement after intravenous administration of contrast media (expression of reduced vascular supply) (Figure 2). Intra-abdominal liquid was present. The walls of the small intestine tract appeared thickened. The left diaphragm was not observable because of the marked intestinal overdistention that misplaced both the heart and the mediastinum (Figure 3). Sub-umbilical laparocele was confirmed (Figure 4). Reactive right pleural effusion with right lung atelectasis was noted. No signs of abdominal free air were observed.

After stabilizing the patient clinically, an emergency laparotomy was performed in relation to the data provided by the CT examination. This approach was selected because of the important distension of the intestinal loops did not allow sufficient space for a complete exploration.

In relation to the previous surgery, the patient presented an important adhesion picture that resulted in obstruction of the small intestine, resolved with adhesiolysis; however, the impairment of the intestinal tract required the execution of a resection with primary ileal anastomosis.

The ischemic suffering also led to the execution of a splenectomy.

The postoperative course was uneventful; patient was discharged at six postoperative days.

At a 2-year follow-up, our patients recovered well, with no further abdominal symptoms.

## Discussion

Volvulus is a condition characterized by a rotation of a bowel segment around itself or its mesentery, determining a partial or closed obstruction of its lumen. Usually, volvulus involves the sigmoid tract of the intestine, the cecum, the stomach and, less frequently, the transverse colon. Small bowel volvulus is rare in adults, being only relatively more frequent in Africa, Asia, the Middle East and India (4, 5). Volvulus is the cause of, approximately, 3–6% of all cases of small bowel obstruction in the western world (6, 7). Associated mortality is high if not promptly diagnosed. The importance to increase physician awareness about its diagnosis cannot be ignored. Small bowel volvulus primary aetiology is defined by volvulus occurring in the absence of anatomical defects, whereas secondary volvulus can occur in presence of anatomical defects, such as bowel malrotation or other anomalies (8). Moreover, secondary volvulus has often been attributed to Meckel’s diverticulum, leiomyomas of the mesentery, adhesions, neoplasms and previous abdominal surgery (9). Small bowel volvulus usually causes acute and severe abdominal pain, often associated with nausea and vomiting. Splenic ischemia is the result of arterial or venous impairment, it is associated with a heterogeneous group of diseases, such as embolism, hematological disorders, malignancies, vasculitis, systemic infection, and trauma, with the ileal volvulus being only a rare cause. There are no tests to exclude strangulated small bowel obstruction and explorative laparotomy should not be delayed. Small bowel volvulus, even if a rare condition, must be considered in patients with sudden onset of acute abdominal pain. Splenic ischemia has to be considered in patients with ileal volvulus and contrasted CT scan must be performed in these patients to confirm the reduction of vascular supply.

## Data Availability

The raw data supporting the conclusions of this article will be made available by the authors, without undue reservation.
